# Supplementary material for: Over-exploitation of natural resources is followed by inevitable declines in economic growth and discount rate
Source: Nat Commun. 2019 Mar 29;10:1419. doi: 10.1038/s41467-019-09246-2 (PMC6440997; doi:10.1038/s41467-019-09246-2)
Supplement: Supplementary file 1 — Supplementary Information [file 41467_2019_9246_MOESM1_ESM.pdf]

Supplementary Information

Over-exploitation of natural resources is followed by inevitable declines in economic growth and discount rate

Adam Lampert

Supplementary Note 1. Discounting with two goods

In this Supplementary Note, we consider a social welfare that is a function of the provision of two goods over time, $f(t)$ and $c(t)$ (e.g., a natural resource and a manufactured good), and we derive the formulas for the discount rate and for the prices of the two goods. We consider the general case in which social welfare, U^T, is given by the standard form given in Eq. 4 (main text), where $u(c, f)$ is twice differentiable with respect to both c and f. We consider a small, marginal perturbation that may vary over time and, at time t, it adds to society $B(t)/\epsilon$ units to some currency, each of which is used to consume $\mu\epsilon$ units of the natural resource, and $(1-\mu)\epsilon$ units of the manufactured good at time t. Consequently, the consumption of the two goods over time becomes

$$c(t) \to c^*(t) = c(t) + (1-\mu)B(t), \tag{A1a}$$

$$f(t) \to f^*(t) = f(t) + \mu B(t). \tag{A1b}$$

We assume that the perturbation is marginal, namely, $(1-\mu)B(t) \ll c(t)$ and $\mu B(t) \ll f(t)$ at all t. Following this perturbation, social welfare becomes

$$\begin{aligned} U^T &= \int_0^T u(c^*, f^*)e^{-\rho t}dt \qquad \text{(A2)} \\ &= \int_0^T u(c, f)e^{-\rho t}dt + \int_0^T \frac{du}{dc}(1-\mu)B(t)e^{-\rho t}dt + \int_0^T \frac{du}{df}\mu B(t)e^{-\rho t}dt. \end{aligned}$$

We denote

$$w(t) = (1-\mu)\frac{\partial u}{\partial c} + \mu\frac{\partial u}{\partial f}, \tag{A3}$$

which implies

$$U^T = U_0^T + \int_0^T B(t)w(t)e^{-\rho t}dt = U_0 + \int_0^\infty B(t)\exp\left(\int_0^t \frac{dw(t')}{dt'}dt' + \rho t\right), \tag{A4}$$

where U_0 is the utility without the perturbation. It follows that the cumulative discount is given by

$$\Delta(t) = -\ln\left(\frac{w(t)}{w(0)}\right) + \rho t. \tag{A5}$$

In turn,

$$\frac{dw}{dt} = (1-\mu)u_{cc}\frac{dc}{dt} + (1-\mu)u_{cf}\frac{df}{dt} + \mu u_{fc}\frac{dc}{dt} + \mu u_{ff}\frac{df}{dt}, \tag{A6}$$

where subscripts of u denote partial derivatives, and the discount rate is given by

$$\begin{aligned} \delta(t) &= \frac{d\Delta}{dt} = -\frac{1}{w}\frac{dw}{dt} + \rho \\ &= -\frac{(1-\mu)u_{cc}\frac{dc}{dt} + (1-\mu)u_{cf}\frac{df}{dt} + \mu u_{fc}\frac{dc}{dt} + \mu u_{ff}\frac{df}{dt}}{(1-\mu)u_c + \mu u_f} + \rho. \qquad \text{(A7)} \end{aligned}$$

Note that several authors suggested one discount rate for the manufactured good and a second discount rate for the natural good[37]. These two discounts are equivalent to Eq. (A7) in the two special cases where $\mu = 0$ and where $\mu = 1$, respectively.

Next, we derive formulas for the changes in the prices of the two goods over time. Note that the prices are well-defined in a perfectly competitive market in which the goods are being traded as long as it increases social welfare, and therefore, at any time t, the market price of c, $P_{\rm c}(t)$, and the market price of f, $P_{\rm f}(t)$, are proportional to the respective derivatives of u at time t:

$$\frac{P_{\rm c}(t)}{P_{\rm f}(t)} = \frac{u_c(t)}{u_f(t)}. \tag{A8}$$

Another constraint that is satisfied by the prices is that the same currency is being used at all times (no inflation), which implies that, for all t,

$$(1-\mu)P_{\rm c}(t) + \mu P_{\rm f}(t) = 1/\epsilon. \tag{A9}$$

In turn, Eqs. A8 and A9 imply

$$P_{\rm c} = \frac{1}{\epsilon}\left[\mu\frac{u_f}{u_c} + (1-\mu)\right]^{-1}, \tag{A10a}$$

$$P_{\rm f} = \frac{1}{\epsilon}\left[(1-\mu)\frac{u_c}{u_f} + \mu\right]^{-1}. \tag{A10b}$$

Also, using the same currency units (in which the weight of the natural resource is given by μ), the total product is proportional to the value of all products at time t, i.e.,

$$\text{product} \sim cP_{\rm c} + fP_{\rm f}. \tag{A11}$$

To calculate $P_{\rm c}$, consider a perturbation that affects only c ($\tilde{\mu} = 0$). Substituting into Eq. A6 implies

$$-\frac{1}{\tilde{w}}\frac{d\tilde{w}}{dt} + \rho = -\frac{u_{cc}\frac{dc}{dt} + u_{cf}\frac{df}{dt}}{u_c} + \rho. \tag{A12}$$

In turn, this must be equal to the rate at which c is discounted[37], which must equal the market discount rate times the rate of change in the price of c:

$$\delta + \nu_{\rm c} = -\frac{u_{cc}\frac{dc}{dt} + u_{cf}\frac{df}{dt}}{u_c} + \rho, \tag{A13}$$

where

$$\nu_{\rm c} \equiv \frac{1}{P_{\rm c}}\frac{dP_{\rm c}}{dt}, \tag{A14}$$

or

$$P_{\rm c}(t) = P_{\rm c}(0)\exp\left(\int_0^t \nu_{\rm c}(t')dt'\right). \tag{A15}$$

Similarly, the change in the price of the natural resource,

$$\nu_{\mathrm{f}} \equiv \frac{1}{P_{\mathrm{f}}}\frac{dP_{\mathrm{f}}}{dt}, \tag{A16}$$

can be calculated by considering a perturbation with $\tilde{\mu} = 1$, which implies

$$\delta + \nu_{\mathrm{f}} = -\frac{u_{ff}\frac{df}{dt} + u_{fc}\frac{dc}{dt}}{u_f} + \rho. \tag{A17}$$

Also, deriving both sides of Eq. A9 with respect to t and substituting Eq. A8 implies

$$\frac{\nu_{\mathrm{c}}}{\nu_{\mathrm{f}}} = -\frac{\mu u_f}{(1-\mu)u_{\mathrm{c}}}. \tag{A18}$$

Finally, note that the changes in the present values of the goods at a given time, which are given by $\delta + \nu_{\mathrm{f}}$ for the natural resource and $\delta + \nu_{\mathrm{c}}$ for the manufactured good, do not depend on μ (Eqs. A13, A17).

Supplementary Note 2. Sustainable discount rates and relative prices

In this Supplementary Note, we calculate the discount rate that emerges when harvest is sustainable, δ_{sus}, as well as the rates of changes in the prices, ν_{c} and ν_{f}, (Eqs. A14, A16) in two special cases. Specifically, we focus on the the asymptotic rates in which the entire system is under sustainable harvest, which implies that c and f increase exponentially (as in other related studies[32,37]):

$$\frac{dc}{dt} = cg_{\mathrm{c}}, \tag{B1a}$$

$$\frac{df}{dt} = fg_{\mathrm{f}}. \tag{B1b}$$

In what follows, we calculate the discount rate in the two special cases: where the goods are non-substitutable, and where the goods are partially-substitutable. Note that similar derivations of δ_{sus} for other forms of the utility function can be found in the literature[32,36,37].

Non-substitutable goods (separable utility function)

For non-substitutable goods, we use the standard, separable utility function given by[12]

$$u(c,f) = (1-\gamma)\frac{c^{1-\eta}}{1-\eta} + \gamma\frac{f^{1-\eta}}{1-\eta}, \tag{B2}$$

where γ is the relative significance of f, and η is the elasticity of utility with respect to consumption, characterizing how fast an increase in utility diminishes. In turn, note that the second partial derivatives of u are given by $u_{cf} = u_{fc} = 0$, $u_{cc} = -\eta u_c/c$, and $u_{ff} = -\eta u_f/f$. Substitution of these partial derivatives into Eq. A7 implies

$$\delta_{\text{sus}} = \frac{(1-\mu)\eta g_{\text{c}} u_c + \mu \eta g_{\text{f}} u_f}{(1-\mu)u_c + \mu u_f} + \rho. \tag{B3}$$

Specifically, if both goods grow exponentially at the same rate, $g_{\text{f}} = g_{\text{c}}$, then

$$\delta_{\text{sus}} = \delta_{\text{today}} = \eta g_{\text{c}} + \rho, \tag{B4}$$

which retrieves Ramsey's discount formula[14,16]. Otherwise, if $0 \leq g_{\text{f}} < g_{\text{c}}$, then substitution of $u_c = (1-\gamma)c^{-\eta}$ and $u_f = \gamma f^{-\eta}$, into Eq. B3 implies

$$\delta_{\text{sus}} = \frac{\eta g_{\text{c}}(1-\mu)(1-\gamma)c^{-\eta} + \eta g_{\text{f}} \mu \gamma f^{-\eta}}{(1-\mu)(1-\gamma)c^{-\eta} + \mu \gamma f^{-\eta}} + \rho. \tag{B5}$$

Specifically, in the limit $t \to \infty$, if $g_{\text{c}} > g_{\text{f}}$ and $\eta > 1$, then $f \ll c$ and $c^{-\eta} \ll f^{-\eta}$. Therefore, if $\eta > 1$ and $\mu > 0$, then

$$\delta_{\text{sus}} \to \eta g_{\text{f}} + \rho \tag{B6}$$

and

$$\delta_{\text{today}} - \delta_{\text{sus}} \to \eta(g_{\text{c}} - g_{\text{f}}) \tag{B7}$$

as $t \to \infty$.

Finally, to calculate the changes in the prices, note that Eq. A13 implies

$$\delta + \nu_{\text{c}} = \eta g_{\text{c}} + \rho. \tag{B8}$$

Specifically, if $\delta = \delta_{\text{sus}}$ and $g_{\text{f}} = g_{\text{c}}$, then $\nu_{\text{c}} = \nu_{\text{f}} = 0$, whereas if $g_{\text{f}} < g_{\text{c}}$ and $t \to \infty$, then

$$\nu_{\text{c}} \to \eta(g_{\text{c}} - g_{\text{f}}) \,, \tag{B9a}$$
$$\nu_{\text{f}} \to 0 \,. \tag{B9b}$$

Partially-substitutable goods (non-separable utility function)

For partially-substitutable goods, we consider a non-separable utility function[12],

$$u(c, f) = \frac{(c^{1-\gamma} f^{\gamma})^{1-\eta}}{1-\eta}, \tag{B10}$$

where, as with the separable utility function, $0 < \gamma < 1$ is the relative significance of f, and η is the elasticity of utility with respect to consumption. Note that the partial derivatives

of u are given by $u_c = (1-\gamma)(1-\eta)u/c$ and $u_f = \gamma(1-\eta)u/f$. In turn, the second partial derivatives of u are given by $u_{cc} = (-\gamma-\eta+\gamma\eta)u_c/c$, $u_{ff} = (\gamma-1-\gamma\eta)u_f/f$ and $u_{cf} = u_{fc} = (1-\gamma)(1-\eta)u_f/c = \gamma(1-\eta)u_c/f$. Substitution of the second partial derivatives in Eq. A7 implies

$$\begin{aligned}\delta_{\text{sus}} &= -\{(1-\mu)u_c\left[(-\gamma-\eta+\gamma\eta)g_{\text{c}}+\gamma(1-\eta)g_{\text{f}}\right] \\ &+ \mu u_f\left[(1-\gamma)(1-\eta)g_{\text{c}}+(\gamma-1-\gamma\eta)g_{\text{f}}\right]\}/\{(1-\mu)u_c+\mu u_f\}+\rho. \quad \text{(B11)}\end{aligned}$$

In turn, substitution of u_c and u_f into Eq. B11 and multiplying both the numerator and the denominator by cf/u implies

$$\begin{aligned}\delta_{\text{sus}} &= -\{(1-\gamma)(1-\mu)f\left[(-\gamma-\eta+\gamma\eta)g_{\text{c}}+\gamma(1-\eta)g_{\text{f}}\right] \\ &+ \gamma\mu c\left[(1-\gamma)(1-\eta)g_{\text{c}}+(\gamma-1-\gamma\eta)g_{\text{f}}\right]\}/\{(1-\mu)(1-\gamma)f+\mu\gamma c\}+\rho.\end{aligned}$$

Some algebra (collecting terms that are identical in both square brackets) implies

$$\delta_{\text{sus}} = \eta g_{\text{c}} + (g_{\text{c}}-g_{\text{f}})\gamma(1-\eta) - (g_{\text{c}}-g_{\text{f}})\frac{\gamma\mu c}{(1-\gamma)(1-\mu)f+\gamma\mu c}+\rho. \quad \text{(B12)}$$

Note that, in the special case where $g_{\text{f}} = g_{\text{c}}$, we obtain Ramsey's discount formula, $\delta_{\text{today}} = \eta g_{\text{c}} + \rho$. If $g_{\text{f}} < g_{\text{c}}$ and $t \to \infty$, then $f \ll c$, and it follows that

$$\delta_{\text{sus}} \to \eta g_{\text{c}} + (\gamma(1-\eta)-1)(g_{\text{c}}-g_{\text{f}}) + \rho \quad \text{(B13)}$$

and

$$\delta_{\text{today}} - \delta_{\text{sus}} \to (\gamma(\eta-1)+1)(g_{\text{c}}-g_{\text{f}}). \quad \text{(B14)}$$

Finally, to calculate the rate of change in prices, note that

$$\begin{aligned}\nu_{\text{c}} &= -\delta - \frac{1}{\tilde{w}}\frac{d\tilde{w}}{dt} + \rho = -\delta - \left[u_{cc}\frac{dc}{dt} + u_{cf}\frac{df}{dt}\right]\Big/ u_c + \rho \\ &= -\delta - (-\gamma-\eta+\gamma\eta)g_{\text{c}} - \gamma(1-\eta)g_{\text{f}} + \rho \\ &= -\delta + \eta g_{\text{c}} + \gamma(1-\eta)(g_{\text{c}}-g_{\text{f}}) + \rho. \quad \text{(B15)}\end{aligned}$$

Similarly,

$$\begin{aligned}\nu_{\text{f}} &= -\delta - \frac{1}{\tilde{w}}\frac{d\tilde{w}}{dt} + \rho = -\delta - \left[u_{ff}\frac{df}{dt} + u_{fc}\frac{dc}{dt}\right]\Big/ u_f + \rho \\ &= -\delta - (1-\gamma)(1-\eta)g_{\text{c}} - (\gamma-1-\gamma\eta)g_{\text{f}} + \rho \\ &= -\delta + \eta g_{\text{c}} + (\gamma-1-\gamma\eta)(g_{\text{c}}-g_{\text{f}}) + \rho. \quad \text{(B16)}\end{aligned}$$

Specifically, note that, if $\delta = \delta_{\text{sus}}$ and $g_{\text{f}} = g_{\text{f}}$, then $\nu_{\text{c}} = \nu_{\text{f}} = 0$, whereas if $g_{\text{f}} < g_{\text{c}}$ and $t \to \infty$, then

$$\nu_{\text{c}} \to g_{\text{f}} - g_{\text{c}}\,, \quad \text{(B17a)}$$
$$\nu_{\text{f}} \to 0\,. \quad \text{(B17b)}$$

Supplementary Note 3. Proof of the theorem

In this Supplementary Note, we prove the theorem (Theorem in the main text). We begin with proving six Lemmas and three Corollaries.

Lemma 1. Assume that $u(c, f)$ is monotonically increasing, twice differentiable with respect to both c and f, and satisfies $u_{ff} < 0$ and $u_{cf} \leq 0$. Consider a perturbation such that $f(t_0)$ increases at a given time t_0, while $c(t_0)$ does not change. Then, the cumulative discount at time t_0, $\Delta(t_0)$, which is given by Eqs. A5, A3 where $0 < \mu \leq 1$, increases due to the perturbation.

Proof of Lemma 1. According to Eq. A5, the discount increases as w decreases. Therefore, we need to show that w (given by Eq. A3) decreases as f increases. Specifically,

$$\frac{dw}{df} = (1-\mu)u_{cf} + \mu u_{ff} \tag{C1}$$

($0 < \mu \leq 1$). In turn, the assumptions that $u_{ff} < 0$ and $u_{cf} < 0$ imply that $dw/df \leq 0$, which complete the proof of Lemma 1. □

Lemma 2A. Assume that $u(c, f)$ is monotonically increasing and twice differentiable with respect to both c and f, where c is given by Eq. 9 and f is given by Eq. 6. Also assume that, as $c \to \infty$ while f remain fixed, $u_{cc}/u_{ff} \to 0$ and $u_{fc}/u_{ff} \to 0$. Consider a perturbation that occurs at a given time t_0, increases $f(t_0)$ by H_0, and decreases $c(t_0)$ by $K(t_0)$, where $0 \leq K(t) \leq K_{\max}$ at all t. Then, for sufficiently large t_0 and sufficiently small H_0, $\Delta(t_0)$ (Eqs. A5, A3) increases due to the perturbation. Specifically, if $g_{\rm f} = 0$ and C_1 and C_2 are bounded from above, then, for sufficiently large t, $\Delta(t)$ increases as the total harvest, $\hat{H} = \alpha H_{\rm s} + H_{\rm n}$, increases (regardless of whether the increase in $\hat{H}$ is due to an increase in $H_{\rm n}$ or $H_{\rm s}$).

Proof of Lemma 2A. To show that Δ increases with $\hat{H}$ if $\hat{H}$ is sufficiently small and t is sufficiently large, we need to show that $dw(t_0)/d\hat{H} < 0$. In turn, it follows from Eq. A3 that

$$\frac{dw}{d\hat{H}} = \frac{dw}{df}\frac{df}{d\hat{H}} + \frac{dw}{dc}\frac{dc}{d\hat{H}} = (\mu u_{ff} + (1-\mu)u_{cf})\beta_0 + (\mu u_{fc} + (1-\mu)u_{cc})\frac{dc}{d\hat{H}}. \tag{C2}$$

Note that, since K is bounded from above, $dc/d\hat{H}$ is negative and is bounded from below, regardless of the values of $H_{\rm n}$ and $H_{\rm s}$ that determine $\hat{H}$. Specifically, there exists $K_{\max} > 0$ such that $dc/d\hat{H} \geq -K_{\max}$. Therefore, it remains to show that, when $t \to \infty$, for any $K_{\max} > 0$,

$$\mu u_{ff} + (1-\mu)u_{cf} \leq K_{\max}(\mu u_{fc} + (1-\mu)u_{cc}), \tag{C3}$$

or, equivalently,

$$\frac{\mu u_{fc} + (1-\mu)u_{cc}}{\mu u_{ff} + (1-\mu)u_{cf}} < \frac{\beta_0}{K_{\max}}. \tag{C4}$$

In turn, Eq. C4 follows from the assumption that

$$\lim_{t\to\infty} \frac{u_{fc}}{u_{ff}} = \lim_{t\to\infty} \frac{u_{cc}}{u_{ff}} = 0. \tag{C5}$$

Specifically, this applies to increasing harvest as this is a special case where $K(t) = \max\{C_1(x_1(t)), C_2(x_2(t))\}$, which completes the proof of Lemma 2A. □

Lemma 2B. Assume that $u(c, f)$ is monotonically increasing and twice differentiable with respect to both c and f, where c is given by Eq. 9 and f is given by Eq. 6. Also assume that, $u_{cc} < 0$, $u_{fc} < 0$, and for all f_0, if $c(t) = c_0 \exp(g_c t)$ and $f(t) = f_0 \exp(g_f t)$, then

$$\lim_{t\to\infty} (\mu u_{ff} + (1-\mu) u_{cf}) e^{g_f t} < 0. \tag{C6}$$

Consider a perturbation that occurs at a given time t_0 and increases $f(t_0)$ by $H_0 e^{g_f}$ and decreases $c(t_0)$ by $K(t)$, where $0 \le K(t) \le K_{\max}$ at all t. Then, for sufficiently large t and sufficiently small H_0, $\Delta(t)$ (Eqs. A5, A3) increases with H_0. Specifically, this implies that if C_1 and C_2 are bounded from above, then, for sufficiently large t, $\Delta(t)$ increases as total harvest, $\hat{H} = \alpha H_s + H_n$, increases (regardless of whether the increase in $\hat{H}$ is due to an increase in H_n or H_s).

Proof of Lemma 2B. As in Lemma 2A, we need to show that, for sufficiently large t_0, $dw(t_0)/d\hat{H} < 0$. In turn,

$$\frac{dw}{d\hat{H}} = \frac{dw}{df}\frac{df}{d\hat{H}} + \frac{dw}{dc}\frac{dc}{d\hat{H}} = (\mu u_{ff} + (1-\mu)u_{cf})e^{g_f t} + (\mu u_{fc} + (1-\mu)u_{cc})\frac{dc}{d\hat{H}}. \tag{C7}$$

Since $u_c > 0$ (u is monotonically increasing) and decreasing with both c and f ($u_{fc} < 0$ and $u_{cc} < 0$), and since $dc/d\hat{H}$ is bounded, it follows that

$$\lim_{t\to\infty} (\mu u_{fc} + (1-\mu)u_{cc}) \frac{dc}{d\hat{H}} = 0. \tag{C8}$$

Therefore, Eq. C6 implies that, for sufficiently large t, $dw/d\hat{H} > 0$. Specifically, this applies to increasing harvest as this is a special case where $K(t) = \max\{C_1(x_1(t)), C_2(x_2(t))\}$, which completes the proof. □

Lemma 3. Assume that $u(c, f)$ is monotonically increasing and twice differentiable with respect to both c and f, where c is given by Eq. 9 where C_1 and C_2 are bounded from above and f is given by Eq. 6. Also assume that, for all f_0, if $c(t) = c_0 \exp(g_c t)$ and $f(t) = f_0 \exp(g_f t)$, then

$$\lim_{t\to\infty} \frac{u_c}{u_f} e^{-g_f t} = 0. \tag{C9}$$

Note that, as a special case where $g_f = 0$, Eq. C9 becomes

$$\lim_{c\to\infty} \frac{u_c}{u_f} = 0. \tag{C10}$$

Then, for sufficiently large t_0, a sufficiently small increase in harvest at t_0, either sustainable or non-sustainable, results in an increase in $u(t_0)$.

Proof of Lemma 3. We need to show that, for any level of the total harvest, $\hat{H} = \alpha H_\text{s} + H_\text{n}$, there exists t', such that increasing $\hat{H}$ would increase u for any $t > t'$. Increasing harvest increases u if and only if $du/d\hat{H} > 0$, and therefore, we need to show that

$$\lim_{t\to\infty} \frac{du}{d\hat{H}} > 0. \tag{C11}$$

Note that,

$$\frac{du}{d\hat{H}} = \frac{du}{df}\frac{df}{d\hat{H}} + \frac{du}{dc}\frac{dc}{d\hat{H}} = u_f \beta_0 e^{g_\text{f} t} + u_c \frac{dc}{d\hat{H}}. \tag{C12}$$

Since C_1 and C_2 are bounded from above, $dc/d\hat{H}$ is bounded from below, namely, there exists $M > 0$ such that $dc/d\hat{H} > -M$. Therefore, to complete the proof, we need to show that, for sufficiently large t,

$$\beta_0 u_f e^{g_\text{f} t} - M u_c \geq 0, \tag{C13}$$

or, equivalently,

$$\frac{u_c}{u_f} e^{-g_\text{f} t} < \frac{\beta_0}{M} \tag{C14}$$

for any $M > 0$. However, this follows directly from Eq. C9, which completes the proof of Lemma 3. □

Corollary 1. Assume that social welfare, U^T, is given by Eq. 4, where ρ is a constant and u follows the assumptions of Lemma 3. Then, optimal harvest dictates that, for sufficiently large t, the entire area is under harvest, namely, $H_\text{n}(t) + H_\text{s}(t) = x_1(t) + x_2(t)$.

Proof of Corollary 1. This Corollary follows directly from Lemma 3. Specifically, increasing *sustainable* harvest at a sufficiently large t_0 increases $u(t_0)$. Moreover, this increase in sustainable harvest necessarily increases U^T as it does not affect future values of u (sustainable harvest does not affect the dynamics of x_1 and x_2). Therefore, optimal harvest dictates that harvest increases until it hits the constraint where $H_\text{n} + H_\text{s} = x_1 + x_2$. This completes the proof of Corollary 1. □

Corollary 2. Assume that social welfare, U^T, is given by Eq. 4, where ρ is a constant and u follows the assumptions of Lemma 3. Then, following the market dynamics, there exists a time t' after which all shared resources are exhausted ($x_2(t) = 0$ for all $t > t'$).

Proof of Corollary 2. Lemma 3 implies that, for sufficiently large t, an increase in harvest increases u. Specifically, since $\alpha < 1$, increasing non-sustainable harvest of the shared resource at time t_0 increases $u(t_0)$, even if it comes on the account of sustainable harvest (and even if harvest non-sustainably is more expensive, $\lambda < 1$). (Note that the non-sustainable harvest may

decrease the future values of u, and therefore, may not be preferable using optimal harvest; however, the managers in the market solution over-harvest despite the future reduction in u due to the non-sustainable harvest of the shared resource.] Therefore, for sufficiently large t, each manager is better off harvesting the shared resource non-sustainably. Thus, there exists t' such that the entire shared resource is being exhausted. This completes the proof of Corollary 2. $\square$

Lemma 4. Assume that social welfare, U^T, is given by Eq. 4, f is given by Eq. 6, c is given by Eq. 9 where C_1 and C_2 are constants, and H_n and H_s are non-negative and satisfy the constraints given by Eqs. 7, 8. Assume that, for sufficiently large t, both cu_{fc}/u_f and fu_{ff}/u_f are monotone with t. Also assume that $u(c, f)$ is non-decreasing in both c and f and that $u_{fc} \leq 0$ and $u_{ff} \leq 0$. Furthermore, assume that $H_\mathrm{n} \geq 0$ and $H_\mathrm{s} \geq 0$ are subject to the constraints given by Eqs. 7, 8 and follow optimal harvest. Then, there exists t' such that if $x(t') = x_1(t') + x_2(t')$ increases, then total harvest, $\hat{H} = H_\mathrm{n} + \alpha H_\mathrm{s}$ increases at all $t > t'$. Namely, for sufficiently large t', if $H^{\mathrm{opt}}_{\mathrm{small}}(t)$ denotes the total optimal harvest in a system with a given $x(t') = x_{\mathrm{small}}$, and $H^{\mathrm{opt}}_{\mathrm{large}}(t)$ denotes the optimal harvest in a system that is identical except that $x(t') = x_{\mathrm{large}} > x_{\mathrm{small}}$, then $H^{\mathrm{opt}}_{\mathrm{large}}(t) \geq H^{\mathrm{opt}}_{\mathrm{small}}(t)$ for all $t > t'$.

Proof of Lemma 4. The idea behind the proof is to show that, for sufficiently large t, $\hat{H} = H_\mathrm{n} + \alpha H_\mathrm{s}$ is non-increasing with time. Since U^T is time invariant when $T \to \infty$, the optimal harvest, $\hat{H}^{\mathrm{opt}}$, depends on time only implicitly via the state variable x ($\hat{H}^{\mathrm{opt}} = \hat{H}^{\mathrm{opt}}(x(t))$). Also, x is non-increasing with time (Eq. 7). Therefore, if $\hat{H}^{\mathrm{opt}}$ decreases with time, this implies that $\hat{H}^{\mathrm{opt}}$ decreases with x.

More formally, note that Corollary 2 implies that, for sufficiently large t, $H_\mathrm{n} + H_\mathrm{s} = x$, and therefore,

$$\hat{H} = H_\mathrm{n} + \alpha H_\mathrm{s} = H_\mathrm{n} + \alpha(x - H_\mathrm{n}) = (1-\alpha)H_\mathrm{n} + \alpha x, \tag{C15}$$

where H_n and H_s denote optimal non-sustainable harvest and optimal sustainable harvest, respectively. In turn, substituting Eq. C15 into Eq. 6 implies

$$f(t) = \hat{H}\beta_0 e^{g_\mathrm{f} t} = [(1-\alpha)H_\mathrm{n} + \alpha x]\beta_0 e^{g_\mathrm{f} t}, \tag{C16}$$

and therefore,

$$\frac{1}{f}\frac{df}{dt} = g_\mathrm{f} + \frac{(1-\alpha)\frac{dH_\mathrm{n}}{dt} - \alpha H_\mathrm{n}}{(1-\alpha)H_\mathrm{n} + \alpha x}, \tag{C17}$$

where we used $dx/dt = -H_\mathrm{n}$ (Eq. 7).

Next, note that postponing one unit of harvest by a small (infinitesimal) unit of time, from t_0 to $t_0 + dt$, implies that one harvests αdt at t_0 plus 1 at $t_0 + dt$ instead of 1 at t_0. When H_n is positive at both t_0 and $t_0 + dt$, it implies that the increase in utility due to extra 1 at t_0 equals an increase in utility due to extra $1 + \tilde{\delta}$ at $t_0 + dt$, where $\tilde{\delta}$ is the rate by which the value of a unit of

fish increases over time. Therefor, either (i) $\delta < \alpha$ and $H_{\rm n} = 0$ or (ii) $H_{\rm n}(t) > 0$ and $\tilde{\delta}(t) = \alpha$:

$$\begin{cases} \tilde{\delta} = \alpha & \text{if } H_{\rm n} > 0 \\ \tilde{\delta} < \alpha & \text{if } H_{\rm n} = 0. \end{cases} \tag{C18}$$

In turn, $\tilde{\delta}$ it is given by the market discount plus the rate of increase in the price of f, $\tilde{\delta} = \delta + \nu_{\rm f}$, which is given by Eq. A7 with $\mu = 1$ (Supplementary Note 1):

$$\tilde{\delta} = -\frac{u_{fc}\frac{dc}{dt} + u_{ff}\frac{df}{dt}}{u_f} + \rho. \tag{C19}$$

Equivalently, we can write

$$\tilde{\delta} = A^{\rm f}(t)\frac{1}{f}\frac{df}{dt} + A^{\rm c}(t)\frac{1}{c}\frac{dc}{dt} + \rho \tag{C20}$$

or

$$\frac{1}{f}\frac{df}{dt} = \frac{\tilde{\delta} - A^{\rm c}(t)\frac{1}{c}\frac{dc}{dt}}{A^{\rm f}(t)}, \tag{C21}$$

where we denote

$$A^{\rm c}(t) \;=\; -c\frac{u_{fc}}{u_f}, \tag{C22a}$$

$$A^{\rm f}(t) \;=\; -f\frac{u_{ff}}{u_f}. \tag{C22b}$$

In turn, it follows that

$$\begin{cases} \frac{1}{f}\frac{df}{dt} = g_{\rm f} + A(t) & \text{if } H_{\rm n} > 0 \\ \frac{1}{f}\frac{df}{dt} < g_{\rm f} + A(t) & \text{if } H_{\rm n} = 0, \end{cases} \tag{C23}$$

where

$$A \equiv \frac{\alpha - A^{\rm c}(t)\frac{1}{c}\frac{dc}{dt}}{A^{\rm f}(t)} - g_{\rm f}. \tag{C24}$$

Furthermore, substituting Eq. C17 into Eq. C23 implies

$$\frac{(1-\alpha)\frac{dH_{\rm n}}{dt} - \alpha H_{\rm n}}{(1-\alpha)H_{\rm n} + \alpha x} \le A(t). \tag{C25}$$

Next, note that the limit $A_\infty = \lim_{t\to\infty} A(t)$ exists. Specifically, since $u_{fc} \le 0$, $u_{ff} \le 0$, $u_c \ge 0$ and $u_f \ge 0$, it follows that $A^{\rm c}(t)$ and $A^{\rm f}(t)$ are non-negative. Furthermore, since cu_{fc}/u_f and fu_{ff}/u_f are monotone for sufficiently large t, it follows that the limits of $A^{\rm c}(t)$ and $A^{\rm f}(t)$ exists (might be infinite), and we denote

$$A^{\rm c}_\infty \;=\; \lim_{t\to\infty} A^{\rm c}(t), \tag{C26a}$$

$$A^{\rm f}_\infty \;=\; \lim_{t\to\infty} A^{\rm f}(t). \tag{C26b}$$

Moreover, we first assume that

$$\lim_{t\to\infty}\frac{1}{c}\frac{dc}{dt}=g_{\mathrm{c}}, \tag{C27}$$

and afterward, we find the asymptotic expansion of H_{n} and we show that it is non-increasing as x decreases, and then, we show that Eq. C27 is consistent and indeed follows from the asymptotic expansion of H_{n}. These considerations imply that $A(t)$ has a limit given by

$$A_\infty=\lim_{t\to\infty}A(t)=\frac{g_{\mathrm{c}}A^{\mathrm{c}}_\infty-\alpha}{A^{\mathrm{f}}_\infty}+g_{\mathrm{f}}. \tag{C28}$$

We distinguish the following three cases. First, in the case where $A_\infty<0$, it follows from Eq. C23 that, for sufficiently large t,

$$\frac{1}{f}\frac{df}{dt}<g_{\mathrm{f}}. \tag{C29}$$

In turn, $f(t)$ is given by Eq. 6, which implies that $d\hat{H}/dt\leq 0$ if and only if Eq. C29 holds. Therefore, if $A_\infty<0$ there exists t' such that $d\hat{H}/dt<0$ for all $t>t'$. Second, in the case where $A_\infty>0$, note that a strict equality in Eq. C25 cannot hold forever. Specifically, the equality implies that $dH_n/dt>0$, which cannot hold forever as the resource is limited ($dx/dt=x$ and $H_n\leq x$). This implies that after any t there have to be intervals where $H_{\mathrm{n}}=0$. However, any continuous deviation of H_{n} from zero yields a continuous deviation of the left-hand-side of Eq. C25 from zero, which would still be greater than $A(t)$ for sufficiently large t. On the other hand, any discontinuous deviation would create an effectively infinitely large derivative. Therefore, if $A_\infty<0$, then there exists a t' such that $H_{\mathrm{n}}(t)=0$ (and $d\hat{H}(t)/dt=0$) for all $t>t'$. Third, in the case where $A_\infty=0$, we use the assumption that for sufficiently large t, both cu_{fc}/u_f and fu_{ff}/u_f are monotone with t. This condition implies that there exists a time t', such that $A(t)$ does not switch signs for all $t>t'$ (namely, either (i) $A_\infty<0$ for all $t>t'$ or (ii) $A_\infty\geq 0$ for all $t>t'$). If (i) holds, then, for sufficiently large t, $dH_{\mathrm{n}}/dt=0$ follows from the same considerations as in Case I. If (ii) holds, then, for sufficiently large t, $dH_{\mathrm{n}}/dt<0$ follows from the same consideration as in Case II.

We have seen that, in all three cases, $\hat{H}(x(t))$ decreases with time, and therefore, it decreases with x. It remain to show that the asymptotic expansion of H_{n} where $t\to\infty$ is either $H_{\mathrm{n}}=0$ or is given by $H_{\mathrm{n}}\sim hx$ where h is a constant, and that this implies that Eq. C27 holds. Specifically, we have already seen that, in Case I, $H_{\mathrm{n}}(t)=0$ if t is sufficiently large, and therefore, we restrict attention to the case $A_\infty>0$. First, we derive $H_{\mathrm{n}}(x)=h(x)x$ with respect to t, which implies

$$\frac{dH_{\mathrm{n}}}{dt}=h\frac{dx}{dt}+x\frac{dh}{dt}=-hH_{\mathrm{n}}+x\frac{dh}{dt}=-h^2x+x\frac{dh}{dt}. \tag{C30}$$

Therefore, it follows from Eq. C25 with equality that

$$\frac{\alpha hx+(1-\alpha)h^2x-x\frac{dh}{dt}}{(1-\alpha)hx+\alpha x}=A(t), \tag{C31}$$

or, after reducing both nominator and denominator by a factor x,

$$\frac{\alpha h + (1-\alpha)h^2 - \frac{dh}{dt}}{(1-\alpha)h + \alpha} = A(t). \tag{C32}$$

Next, we look for an asymptotic solution where h is a constant ($dh/dt = 0$):

$$\frac{\alpha h + (1-\alpha)h^2}{(1-\alpha)h + \alpha} = A_\infty. \tag{C33}$$

Namely,

$$(1-\alpha)h^2 + [\alpha - (1-\alpha)A_\infty]h - \alpha A_\infty = 0, \tag{C34}$$

which implies

$$h_{1,2} = \frac{(1-\alpha)A_\infty - \alpha \pm (1-\alpha)A_\infty + \alpha}{2(1-\alpha)}, \tag{C35}$$

or

$$h_1 = A_\infty, \tag{C36a}$$

$$h_2 = -\frac{\alpha}{1-\alpha}, \tag{C36b}$$

and since only $h_1 \geq 0$, we get $h = A_\infty$.

Finally, we need to show that $H_\mathrm{n} = hx$ implies that $(1/c)(dc/dt) \to g_\mathrm{c}$ as $t \to \infty$. Specifically, it follows from Corollary 2 that, for sufficiently large t, $H_\mathrm{n} + H_\mathrm{s} = x$. Substituting this in Eq. 9 implies

$$c = c_0 e^{g_\mathrm{c} t} - C_1(1-\lambda)H_\mathrm{n} - C_2\lambda x], \tag{C37}$$

which yields

$$\frac{dc}{dt} = g_\mathrm{c}\left[c - C_1((1-\lambda)H_\mathrm{n})\right], \tag{C38}$$

or

$$\frac{1}{c}\frac{dc}{dt} = g_\mathrm{c} - \frac{g_\mathrm{c} C_1((1-\lambda)H_\mathrm{n})}{c_0 e^{g_\mathrm{c} t} - C_1\left((1-\lambda)H_\mathrm{n} + \lambda x\right)}. \tag{C39}$$

Substituting $H_\mathrm{n} = hx$ implies

$$\frac{1}{c}\frac{dc}{dt} = g_\mathrm{c} - \frac{g_\mathrm{c} C_1(1-\lambda)hx}{c_0 e^{g_\mathrm{c} t} - C_1\left((1-\lambda)hx + \lambda x\right)}, \tag{C40}$$

or

$$\frac{1}{c}\frac{dc}{dt} = g_\mathrm{c} + \frac{g_\mathrm{c} C_1((1-\lambda)h)}{\frac{c_0 e^{g_\mathrm{c} t}}{x} - C_1\left((1-\lambda)h + \lambda\right)}. \tag{C41}$$

Note that $c_0 \exp(g_\mathrm{c} t)/x \to \infty$ as $t \to \infty$, which implies that $(1/c)(dc/dt) \to g_\mathrm{c}$ as $t \to \infty$. This completes the proof of Lemma 4. □

Corollary 3. Assume that social welfare, U^T, is given by Eq. 4, where ρ is a constant, f is given by Eq. 6 and c is given by Eq. 9 where $C_1 = C_2 =$ constant. Consider two identical

systems with the only difference being that one is subject to market harvest and the other is subject to optimal harvest. Denote x_{market} the value of $x = x_1 + x_2$ that follows from market dynamics and x_{opt} the value of x that follows socially optimal harvest (see Methods). Then, for any t, $x_{\mathrm{opt}} \geq x_{\mathrm{market}}$.

Proof of Corollary 3. Note that the future cost incurred by the harvest of the shared resource to the individual in a perfectly competitive market is 0, whereas the future cost to society is positive as it cannot harvest this resource later (Lemma 3 guarantees that the difference in cost is strictly positive). Therefore, the shared resource may be over-exploited following market dynamics. Therefore, whenever $x_{\mathrm{opt}} = x_{\mathrm{market}}$, market harvest is at least as large as optimal harvest. Since initially $x_{\mathrm{opt}} = x_{\mathrm{market}}$, it follows that $x_{\mathrm{opt}} \geq x_{\mathrm{market}}$ at all t, which completes the proof of Corollary 3. □

Lemma 5. Assume that H_{n} and H_{s} are non-negative and satisfy the constraints given by Eqs. 7, 8, where $H_{\mathrm{n}} = H_{\mathrm{n}}^1 + H_{\mathrm{n}}^2$ and $H_{\mathrm{s}} = H_{\mathrm{s}}^1 + H_{\mathrm{s}}^2$. Assume that $H_{\mathrm{n}}(t) > 0$ during a given time interval, $t \in [t_0, t_1]$. Then, there exists a time $t_{\mathrm{c}} > t_1$, such that the total harvest at time t_{c}, $\hat{H}(t_{\mathrm{c}}) \equiv H_{\mathrm{n}}(t_{\mathrm{c}}) + \alpha H_{\mathrm{s}}(t_{\mathrm{c}})$, satisfies

$$\hat{H}(t_{\mathrm{c}}) < \alpha(x_1(t_0) + x_2(t_0)). \tag{C42}$$

Proof of Lemma 5. Assume that, in contrast to Eq. C42,

$$\hat{H}(t) \geq \alpha(x_1(t) + x_2(t)) \tag{C43}$$

for all $t > t_1$. Denote x_H the total area that has been degraded due to non-sustainable harvest from time t_0 to time t_1,

$$x_{\mathrm{H}} = \int_{t_0}^{t_1} H_{\mathrm{n}}(t)dt. \tag{C44}$$

It follows from Eq. 7 that, for all $t > t_1$,

$$x_1(t) + x_2(t) \leq x_1(t_0) + x_2(t_0) - x_{\mathrm{H}}. \tag{C45}$$

(Strict inequality is possible if $H_{\mathrm{n}}(t) > 0$ at times greater than t_1.) In turn, Eqs. 6 and C45 imply that

$$H_{\mathrm{n}}(t) + H_{\mathrm{s}}(t) \leq x_1(t_0) + x_2(t_0) - x_{\mathrm{H}} \tag{C46}$$

for all $t > t_1$. Next, Eqs. C43 and Eq. C46 imply that

$$H_{\mathrm{n}}(t) \geq \frac{\alpha}{1-\alpha} x_{\mathrm{H}} \tag{C47}$$

for all $t > t_1$. In turn, note that the right-hand-side of Eq. C47 is a constant that does not depend on time, and therefore, it follows from Eqs. 7 and C47 that $x_1(t) + x_2(t)$ is negative for

sufficiently large t. However, this contradicts the Lemma's assumptions because $x_1 + x_2$ must be non-negative at all times to satisfy Eq. 8 where H_n and H_s are non-negative. Therefore, Eq. C43 cannot hold for all $t > t_1$, which completes the proof of Lemma 5. □

Proof of Theorem. The assumptions of the theorem satisfy the assumptions of all Lemmas and corollaries in this Supplementary Note. First, note that, if $C_1 = C_2$ =constant, then it follows from Corollary 3 that $x_\text{opt} \geq x_\text{market}$ at all t. Then, according to Corollary 2, there exists t_1 such that $x_2(t) = 0$ and market follows optimal dynamics for all $t > t_1$. Then, for all $t > t_1$, the only difference between the systems is that $x_\text{opt}(t) \geq x_\text{market}(t)$. Therefore, it follows from Lemma 4 that, for sufficiently large t, harvest in the system that follows optimal harvest is not smaller than harvest in the system that follows market harvest. Then, it follows from Lemmas 2A and 2B that $\Delta_\text{opt} \geq \Delta_\text{market}$, which completes the proof of the first part of the theorem.

Next, denote $\hat{H}_\text{sus}$ the total harvest ($H_\text{n} + \alpha H_\text{s}$) following the optimal sustainable harvest, and denote $\hat{H}_\text{market}$ the total harvest in the case where $H_\text{n}(t) > 0$ from time t_0 to time t_1. Corollary 1 implies that, for sufficiently large t, H_sus is given by the the right-hand-side of Eq. C42 in Lemma 5. Therefore, Lemma 5 implies that, there exists $t_\text{c} > t_1$, such that $\hat{H}_\text{sus}(t_\text{c}) > \hat{H}_\text{market}(t_\text{c})$. This completes the proof of the second part of the theorem. □

Supplementary Note 4. The Stochastic Programming algorithm

In this Supplementary Note, we describe the algorithm that we used for the numerical simulations of the model. First, we describe the algorithm that we used for finding the socially optimal solution (Fig. 2A, B). Then, we describe the algorithm that we used for finding the market solution (Fig. 2C, D). Specifically, to find the optimal solution, we implemented a Stochastic Programming algorithm in C/C++. Here we focus on details that are more specific to the particular model described in the paper. For a more detailed explanation on Stochastic Programming, see Clark & Mangel (2000)[44]

Optimal solution

As in a standard Dynamic Programming method, we first set a terminal time, T. Then, the algorithm uses some heuristics[§] to set the values of $U^*_T(x)$, which are the utilities that the society

[§]The algorithm would work even if we simply set $U^*_T(x) = 0$; however, the algorithm uses the following heuristics that helps it converging faster, and yields the same results when $T \to \infty$: It calculates $U^*_T(x)$ as the social welfare added to the system, assuming that only sustainable harvest takes place for the 1000 years that

would have from still having $x = x_1(T) + x_2(T)$ non-degraded areas when arriving to time T. Specifically, the algorithm calculates $U_T^*(x)$ for all values of x up to some resolution, Δx (namely, $x = 0, \Delta x, 2\Delta x, ..., x_{\max}$, where $x_{\max} = x_1(0) + x_2(0)$). The algorithm chooses a sufficiently small Δx, such that the results well-approximates the a dynamics of a continuous variable ($\Delta x = 10^{-5}$ in Fig. 2A, B). Furthermore, when calculating the optimal solution, the only difference between a private and a public land is the potentially different costs. Therefore, the algorithm incorporates a single state variable, x, where the cost of harvest is given by a single function that incorporates both C_1 and C_2:

$$C_{\text{eff}}(x) = \begin{cases} C_1(x) & \text{if } C_1(x) > C_2(0) \\ C_2(x) & \text{if } C_2(x) > C_1(0) \\ C_1(x_1)|_{C_1(x_1)=C_2(x-x_1)} & \text{otherwise.} \end{cases} \tag{D1}$$

Next, the algorithm goes backward in time and, for each t, it calculates, for each x, the optimal strategy at time $t-1$, using the value of each state at time t that was calculated at the previous step. Specifically, the contribution to the welfare from time $t-1$ onward is given by

$$U_{t-1}(x(t-1), \mathbf{H}) = u\left(c(\mathbf{H}, t-1), f(\mathbf{H}, t-1)\right) + e^{-\rho} U_t^*(x(t)), \tag{D2}$$

where $\mathbf{H}$ is the harvest profile at time t ($\mathbf{H} = (H_{\text{n}}, H_{\text{s}})$), the asterisk indicates that U_t^* is the welfare that results from using the optimal strategy from time t onward. Specifically, $c(\mathbf{H}, t-1)$ is the amount of manufactured good consumed at time $t-1$ and $f(\mathbf{H}, t-1)$ is the amount of natural resource harvested at time $t-1$, where the harvest functions are given by $\mathbf{H}$. In turn, the dynamics of $f(\mathbf{H}, t)$ and $c(\mathbf{H}, t)$ are given by Eqs. 6, 9 (main text), and they depend on $\mathbf{H}(x, t-1)$. Note that $x(t)$ also depends on $\mathbf{H}$, since $x(t) = x(t-1) - H_{\text{n}}(t-1)$ (Eq. 7). Next, for each x, the algorithm finds the harvest profile $\mathbf{H}^*(x, t-1)$, the harvest profile that maximizes $U_{t-1}(x)$. In turn, this implies that $U_{t-1}^*(x(t-1)) = U_{t-1}(x(t-1), \mathbf{H}^*)$. Repeating this backward induction algorithm from $t = T$ back to $t = 0$ provides the entire harvest profile at all times, given a terminal time T. Finally, to find the optimal harvest in the limit where $T \to \infty$, we chose a sufficiently large T such that further increasing T makes no visible change on the numeric results¶. Note that, in the simulations generating Fig. 2, the instantaneous utility function, u, is given either by the separable utility function, Eq. B2 (Fig. 2A), or by the non-separable utility function, Eq. B10 (Fig. 2B).

Market solution

To calculate the solution that emerges following market dynamics, we consider two state variables, $x_1(t)$ and $x_2(t)$, which follow Eq. 7, as described in the main text. For numeric purposes,

follow year T.

¶Note that the value of T in our simulations is greater than the largest value of t that is visible in Fig. 2

these variables are discretized using some fine resolution ($\Delta x_1 = \Delta x_2 = 10^{-3}$ in Fig. 2C, D). As in the algorithm used for finding the optimal solution, we set some terminal time, T, and the algorithm uses the same heuristics to set the terminal values, $U^*_T(x_1, x_2)$, characterizing the value of having $x_1(T)$ private and $x_2(T)$ public non-degraded natural resource at time T. Next, the algorithm goes backward in time and, for each t, it calculates, for each state (characterized by an (x_1, x_2)-pair), the Nash equilibrium at time $t-1$, using the value of each state at time t, which was calculated at the previous step. We consider a perfectly competitive market in which there are infinitely many managers and the actions of a given manager have no influence on her/his future revenues from the shared resource. Therefore, the objective is to find a solution that maximizes social welfare, with the exception that we ignore the externality that emerges via harvesting in the shared regions[12,40].

Specifically, the contribution to the welfare from time $t-1$ onward is given by

$$U_{t-1}(x_1(t-1), x_2(t-1), \mathbf{H}) = u\left(c(\mathbf{H}, t-1), f(\mathbf{H}, t-1)\right) + e^{-\rho} U^*_t(x_1(t), x_2(t)), \quad \text{(D3)}$$

where the notations are similar to those that are used for describing the algorithm that finds the optimal solution, except that here the harvest profile comprises the four harvest functions ($\mathbf{H} = (H^1_{\text{n}}, H^2_{\text{n}}, H^1_{\text{s}}, H^2_{\text{s}})$) and U^*_t is the utility that result from the solution of the market dynamics (i.e., in the Nash equilibrium). The dynamics of the state variables are given by

$$x_1(t) = x_1(t-1) - H^1_{\text{n}}, \quad \text{(D4a)}$$
$$x_2(t) = x_2(t-1) - \tilde{H}^2_{\text{n}}, \quad \text{(D4b)}$$

where consistency implies that $\tilde{H}^2_{\text{n}}$ must be equal to H^2_{n}. Nevertheless, in the market dynamics, each managers ignores her/his own effect on x_2 and considers $\tilde{H}^2_{\text{n}}$ as given and determined by the strategies of the other agents. Therefore, for each (x_1, x_2)-pair, the algorithm finds $\mathbf{H}^*(x_1, x_2, t-1) = (H^{1*}_{\text{s}}, H^{1*}_{\text{n}}, H^{2*}_{\text{s}}, H^{2*}_{\text{n}})$ that maximizes Eq. D3 subject to Eq. D4 for various values of $\tilde{H}^2_{\text{n}}$, until it finds a value of $\tilde{H}^2_{\text{n}}$ for which $\tilde{H}^2_{\text{n}} = H^{2*}_{\text{n}}$. (Specifically, it starts from $\tilde{H}^2_{\text{n}} = 0$, and increases $\tilde{H}^2_{\text{n}}$ gradually while finding each time the optimal solution, until it finds a solution that also satisfies $\tilde{H}^2_{\text{n}} = H^{2*}_{\text{n}}$.) Repeating this backward induction for the times from $t = T$ back to $t = 0$ provides the entire harvest profile at all times, given a terminal time T. Finally, to find the optimal harvest in the limit where $T \to \infty$, we chose a sufficiently large T such that further increasing T makes no visible change on the numeric results (see previous footnote¶).
